# Ocular and neural distribution of feline herpesvirus-1 during active and latent experimental infection in cats

**DOI:** 10.1186/1746-6148-9-185

**Published:** 2013-09-22

**Authors:** Wendy M Townsend, Susan Jacobi, Shih-Han Tai, Matti Kiupel, Annabel G Wise, Roger K Maes

**Affiliations:** 1 Department of Small Animal Clinical Sciences, College of Veterinary Medicine, Michigan State University, D208 Veterinary Medical Center, 48824-1314 East Lansing, MI, USA; 2 Diagnostic Center for Population and Animal Health, Michigan State University, 4125 Beaumont Road, 48910-8104 Lansing, MI, USA; 3 The current address: Department of Veterinary Clinical Sciences, Purdue University, 47907-2026 W. Lafayette, IN, USA; 4 The current address: Animal Eye Care, 1612 Washington Blvd, 94539 Fremont, CA, USA; 5 The current address: National Cancer Institute, Bldg. 535, Room 324, 1050 Boyles St., PO Box B21702 Frederick, MD, USA

**Keywords:** Feline, Felid herpes virus1, FeHV-1, Latency, Ganglia, Eye

## Abstract

**Background:**

Herpes simplex virus 1 (HSV-1) and varicella zoster virus (VZV) cause extensive intra-ocular and neural infections in humans and are closely related to Felid herpes virus 1 (FeHV-1). We report the extent of intra-ocular replication and the extent and morphological aspects of neural replication during the acute and latent phases of FeHV-1 infection. Juvenile, SPF cats were inoculated with FeHV-1. Additional cats were used as negative controls. Cats were euthanized on days 6, 10, and 30 post-inoculation.

**Results:**

FeHV-1 was isolated from the conjunctiva, cornea, uveal tract, retina, optic nerve, ciliary ganglion (CG), pterygopalatine ganglion (PTPG), trigeminal ganglion (TG), brainstem, visual cortex, cerebellum, and olfactory bulb of infected cats during the acute phase, but not the cranial cervical ganglion (CCG) and optic chiasm. Viral DNA was detected in all tissues during acute infection by a real-time quantitative PCR assay. On day 30, viral DNA was detected in all TG, all CCG, and 2 PTPG. Histologically mild inflammation and ganglion cell loss were noted within the TG during acute, but not latent infection. Using linear regression, a strong correlation existed between clinical score and day 30 viral DNA copy number within the TG.

**Conclusions:**

The correlation between clinical score and day 30 viral DNA copy number suggests the severity of the acute clinical infection is related to the quantity of latent viral DNA. The histologic response was similar to that seen during HSV-1 or VZV infection. To the author’s knowledge this is the first report of FeHV-1 infection involving intraocular structures and autonomic ganglia.

## Background

Felid herpesvirus-1 (FeHV-1) is an enveloped dsDNA virus classified under the genus *Varicellovirus* within the subfamily *Alphaherpesvirinae*[1]. In susceptible cats, FeHV-1 accounts for approximately 50-75% of upper respiratory infections [2]. Following exposure of the conjunctival and oronasal mucous membranes, FeHV-1 replicates extensively at these sites, resulting in pyrexia, depression, anorexia, sneezing, conjunctivitis, keratitis, and oculonasal discharge [3]. The acute phase of FeHV-1 infection is followed by lifelong latency [4]. Latency is defined as an asymptomatic period during which infectious virus cannot be recovered from tissues, but can be induced to reactivate through organ culture or explantation [5,6]. Reactivation of a previously latent infection occurs readily in response to natural stressors or administration of corticosteroids [4]. Reactivation is associated with renewed virus production, shedding of infectious virus, and ocular or nasal lesions [4].

In a murine herpes simplex virus 1 (HSV-1) model, the spread of HSV-1 from initial corneal epithelial replication sites to intra-ocular structures and the nervous system has been well documented [7]. After topical inoculation of scarified corneas of adult mice, viral antigen has been detected using immunohistochemistry by day 6 in the cornea, conjunctiva, iris, choroid, retina, ciliary ganglia (CG), trigeminal ganglia (TG), pterygopalatine ganglia (PTPG), superior cervical ganglia, brainstem, olfactory bulb, and hypothalamus [7]. HSV-1 infection has also been documented in human CG [8]. Latent varicella zoster virus (VZV) has been demonstrated in cranial nerve ganglia, dorsal root ganglia, and autonomic nervous system ganglia of human cadavers using polymerase chain reaction (PCR) and *in situ* hybridization [9-12]. The VZV has also been isolated from corneas of humans with acquired immunodeficiency syndrome and chronic keratitis [13]. Previous reports have shown that FeHV-1 can be isolated during the acute phase of the disease from the cornea, conjunctiva, nasal epithelium, nasal turbinates, tonsils, and TG [14,15]. The presence of viral DNA during latency has been demonstrated by PCR in the cornea, nasal turbinates, olfactory bulbs, cerebrum, optic nerve, optic chiasm, and TG [16-18]. Latency associated transcripts (LATs), small strands of RNA transcribed by the virus within latently infected neurons [5,6], have been shown to be present within latently infected TG [18].

The TG or other neural tissues of FeHV-1 infected animals have not been examined histologically to document the lesions caused by FeHV-1 infection. In the TG of mice acutely infected with HSV-1, the virus has been detected within both neural and non-neural cells [19]. Infection with HSV-1 also induced apoptosis of neurons and a histiocytic and lymphocytic infiltrate [20-22]. The TG of mice latently infected with HSV-1 typically demonstrated a mononuclear cellular infiltrate that has also been documented in the TG of latently infected humans [23-28]. This cellular infiltrate is believed to play a critical role in the prevention of recrudescence [29].

In this study we defined the ocular and neural tissues with detectable virus during active and latent FeHV-1 infection and quantified the amount of virus present within the cornea, conjunctiva, TG, CG, PTPG, cranial cervical ganglia (CCG), uvea, retina, optic nerve, optic chiasm, visual cortex, cerebellum, brainstem, and olfactory bulb. The study population consisted of 3 groups each composed of 4 inoculated cats and 1 control cat. Samples were collected from group 1 on day 6 post inoculation (PI) when all FeHV-1 exposed cats were exhibiting ocular signs, from group 2 on day 10 PI which was the peak of ocular and respiratory signs, and from group 3 on day 30 PI when the clinical signs had resolved and latency was established. The TG and CG were examined histologically to detect lesions associated with both active and latent infection. Correlations between the severity of clinical signs and the viral load within the TG were made from samples collected on days 6, 10, and 30 post-inoculation.

## Results

### Clinical scores and serologic evaluation

All inoculated cats developed clinical signs such as sneezing, ocular discharge, nasal discharge, anorexia, and elevated body temperature that were consistent with FeHV-1 infection, whereas all control cats remained disease-free. The median total of the clinical scores from day 0 to the day of euthanasia was 6 (range 4 to 8) for group 1 (day 6 PI), 21 (range 17 to 28) for group 2 (day 10 PI), and 19.5 (range 17 to 32) for group 3 (day 30 PI). In group 3 all clinical signs had resolved prior to euthanasia. The control cats did not develop FeHV-1 specific virus neutralizing (VN) antibodies. The group 3 inoculated cats seroconverted as the FeHV-1 VN antibody titers were less than 1:4 at days 7 and 14 PI and increased to 1:32 to 1:128 at days 21 and 30 PI.

### Virus isolation (VI)

The VI data are presented in Table 1. FeHV-1 was not isolated from any of the samples taken from the control cats. Except for the cranial cervical ganglion, virus was isolated from each of the tissue types collected from group 1 (day 6 PI), group 2 (day 10 PI), or both. Except for a single corneal sample from a cat in group 3 (cat #11), virus was not isolated from any tissue sample collected from group 3 (day 30 PI).

**Table 1:** Virus isolation results

**Tissue**	**Group 1 (Day 6)**	**Group 2 (Day 10)**	**Group 3 (Day 30)**
	**(# pos/ # tested)**	**(# pos/ # tested)**	**(# pos/ # tested)**
Cornea	4/4	4/4	1/4
Conjunctiva	4/4	4/4	0/4
Trigeminal ganglia	2/4	2/4	0/4
Ciliary ganglia	2/4	2/4	0/4
PTPG	4/4	3/4	0/4
CCG	0/4	0/4	0/4
Uvea	4/4	4/4	0/4
Retina	4/4	4/4	0/4
Optic nerve	3/4	1/4	0/4
Optic chiasm	3/4	0/4	0/4
Visual cortex	4/4	1/4	0/4
Cerebellum	3/4	3/4	0/4
Brainstem	3/4	2/4	0/4
Olfactory bulb	4/4	2/4	0/4

Virus isolation results obtained for each tissue type on each day. All control samples were negative and are not listed within this table. *PTPG* pterygopalatine ganglia. *CCG* cranial cervical ganglia.

### RT-PCR

**Figure 1:**
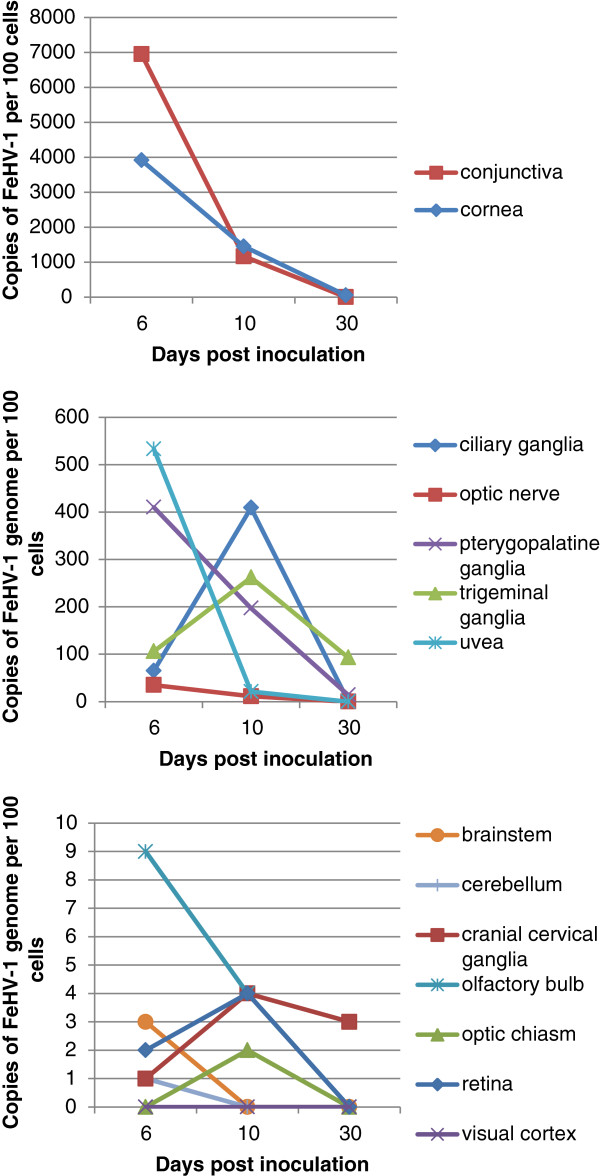
**FeHV-1 copy number versus days post inoculation.** The average number of copies of FeHV-1 genome per 100 cells was plotted against the days post inoculation. The FeHV-1 copy number decreases over time in most tissues sampled. However, the ciliary ganglia, trigeminal ganglia, cranial cervical ganglia, and optic chiasm had higher levels of virus present at day 10 than at day 6.

**Figure 2:**
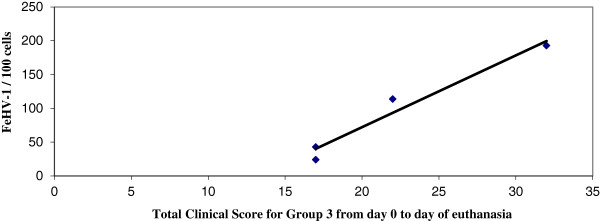
**Clinical score versus FeHV-1 copy number within the trigeminal ganglia (TG).** Linear regression of the total clinical score for group 3 from day 0 to the day of euthanasia compared to the FeHV-1 copy number per 100 cells within the TG. A close linear relationship was seen between the amount of latent virus present within the TG and the total clinical score for each individual animal in group 3. The p-value was 0.021 and R^2^ was 0.9583.

The PCR results for each individual tissue type and the FeHV-1 DNA copy numbers reported per 100 cells of the homogenized tissues examined are shown in Table 2 and Figure 1. As the number of FeHV-1 genomes/μg of sample DNA was known, the FeHV-1 copy number per 100 cells was calculated based on 260,000 haploid genome equivalents/μg of feline cellular DNA [30]. Individual cell types were not examined. The FeHV-1 copy number per 100 cells was reported to standardize comparisons between the various tissues as different tissues yielded different amounts of DNA. FeHV-1 DNA was not detected by real-time PCR in any of the samples obtained from the control cats. For each group, the total of the clinical scores from Day 0 to the day of euthanasia and the FeHV-1 DNA copy number per 100 cells in the TG were compared by linear regression (Figure 2). The total clinical score and FeHV-1 copy number were strongly correlated (p=0.021, R^2^=0.9583) for group 3. The clinical score and FeHV-1 copy number were not correlated for groups 1 (p=0.39, R^2^=0.37) or 2 (p=0.41, R^2^=0.36).

**Table 2:** Real time PCR assay results

	**Group 1 (Day 6)**		**Group 2 (Day 10)**		**Group 3 (Day 30)**	
**Tissue**	RT-PCR	Average FeHV-1 DNA copies/100 cells	RT-PCR	FeHV-1/100 cells	RT-PCR	FeHV-1/100 cells
	(#pos/# tested)		(#pos/# tested)		(#pos/# tested)	
Cornea	4/4	3915	4/4	1450	1/4	49
Conjunctiva	4/4	6950	4/4	1168	1/4	<1
TG	4/4	106	4/4	262	4/4	93
CG	4/4	65	4/4	409	1/4	1
PTPG	4/4	410	4/4	197	2/4	14
CCG	3/4	1	4/4	4	4/4	3
Uvea	4/4	533	4/4	21	0/4	0
Retina	4/4	2	4/4	4	0/4	0
Optic nerve	4/4	35	4/4	11	1/4	<1
Op chiasm	4/4	<1	4/4	2	0/4	0
Vis cortex	4/4	<1	2/4	<1	0/4	0
Cerebellum	4/4	1	4/4	<1	0/4	0
Brainstem	3/4	3	2/4	<1	0/4	0
Olf bulb	4/4	9	4/4	4	0/4	0

Real-time PCR assay (RT-PCR) results obtained for each tissue type on each day. All control samples were negative and are not listed within this table. *TG* trigeminal ganglia. *CG* ciliary ganglia. *PTPG* pterygopalatine ganglia. *CCG* cranial cervical ganglia. *Op chiasm* optic chiasm. *Vis cortex* visual cortex. *Olf bulb* olfactory bulb.

### Histopathology

**Figure 3:**
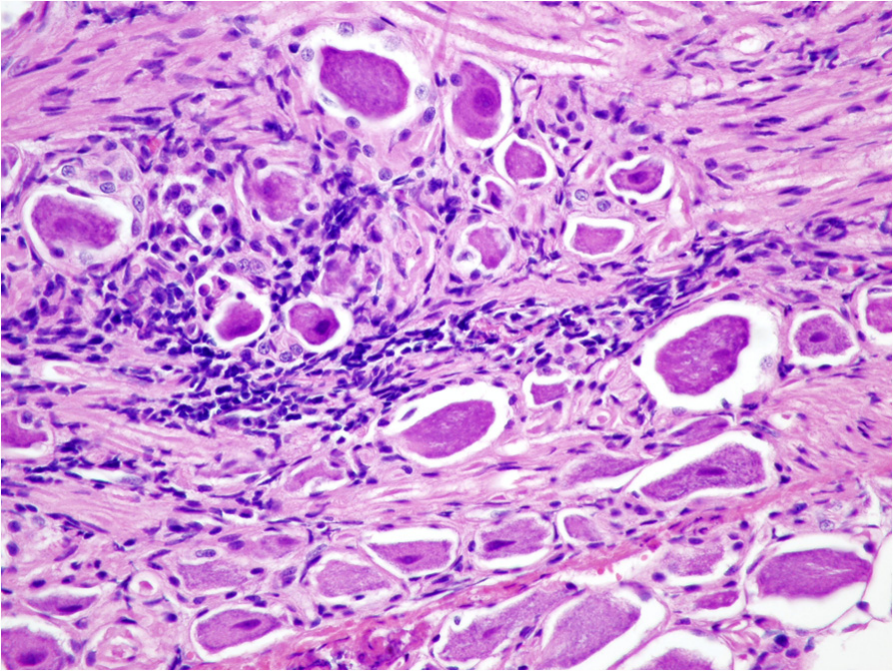
**Histologic section of the trigeminal ganglion.** Hematoxylin and eosin stained section of the trigeminal ganglion taken at 400X magnification. Note the lymphoplasmacytic inflammation surrounding the neurons.

The corneas, conjunctiva, TG, and CG were examined histologically. No histological changes were found within the cornea, conjunctiva, TG, or CG of control animals. No histologic changes were noted within any of the corneal samples except for one cat from group 2. In that sample multiple, solitary, degenerate epithelial cells and a mild lymphocytic infiltrate were noted. Histologic changes consisting of epithelial cell attenuation and/or loss and lymphoplasmacytic inflammation were seen in the conjunctival samples from all 12 inoculated cats. The changes were milder in the group 3 cats. All 4/4 TG obtained from group 1 and 3/4 obtained from group 2 had histologic changes consisting of ganglion cell death, depletion of ganglionic cells, increased numbers of glial cells, and lymphoplasmacytic inflammation (Figure 3). One/four TG obtained from group 3 showed ganglion cell depletion, but no concurrent inflammation. Histologic changes were not evident in the examined CG of inoculated cats. However in 3 cats the small size of the CG precluded obtaining an appropriate section for histologic evaluation.

## Discussion

FeHV-1 infection in cats is a valid natural host model to study herpesvirus-induced ocular disease and latency in humans. In this study we describe intra-ocular and neural FeHV-1 involvement during the acute phase of the infection of tissues (CG, PTPG, CCG, uvea, and retina) from which FeHV-1 has not previously been isolated [14-18]. During the latent phase of infection, latent viral DNA was detected not only within the TG, but also the CCG of all cats and in PTPG of two of the inoculated cats. Morphologically ganglionic cell death and inflammation were present within the TG during the acute phase of infection.

To our knowledge, involvement of the uveal tract during the acute phase of FeHV-1 infection has not previously been documented. Felid herpesvirus-1 DNA has been detected by PCR in the aqueous humor of cats with idiopathic uveitis and in clinically normal cats [31]. However, in previous studies only the uveal tract of latently infected cats has been examined and virus was not detected [17]. The isolation of FeHV-1 from the uveal tract demonstrates that cats, like mice infected with HSV-1 [32], experience active intra-ocular virus replication.

Another not previously reported finding was the isolation of FeHV-1 from all retinas during the acute phase of the disease. As retinal homogenates were used in the current study, we were unable to determine the specific layer of the retina involved during the acute phase of infection. In one murine model HSV-1 study, utilizing corneal scarification prior to inoculation, infectious virus was detected within the retinal photoreceptors [33]. Noteworthy also is that after intracameral administration of HSV-1 in a murine HSV-1 model, the virus is detected within the contralateral, but not the ipsilateral retina [34]. In the present study, FeHV-1 reached the retina without intracameral injection or corneal scarification. No visual deficits were noted. However fundic examinations were not performed as part of this study and therefore retinal abnormalities might not have been detected. In addition, histologic examination was not performed on any of the intra-ocular tissues. Therefore while FeHV-1 was detected within multiple ocular tissues, the morphological changes due to the presence of the virus were not evaluated.

In the murine HSV-1 model an intense inflammatory response is noted histologically in response to the intra-ocular infection [33,34]. In contrast, the cats in this study showed no clinical signs of intraocular inflammation despite the presence of relatively large amounts of replicating virus. Perhaps the magnitude of the infection (8–14 times less virus in the intra-ocular tissues compared with the cornea and conjunctiva) explains the lack of intra-ocular inflammation noted in this study. The amount of FeHV-1 present within the intra-ocular tissues is much less than the 2×10^4^ plaque forming units used to inoculate the anterior chamber in the murine model [34]. Ocular examination with a slit-lamp biomicroscope would be required in future studies to detect subtle inflammation. Future studies combining histologic examination and *in situ* hybridization will further clarify this difference.

Virus particles and FeHV-1 DNA were detected during this study within the optic nerve and optic chiasm. Since these tissues lack neuronal cell bodies [35] the virus must either have been undergoing axonal transport [35], have infected glial cells within the optic nerve and chiasm [36], or have been present within leukocytes within the neural tissues [37]. Reubel et al. [16] and Weigler et al. [17] have previously detected latent FeHV-1 DNA within the optic nerves and chiasms using PCR assays. In this study latent viral DNA was not detected within optic chiasm homogenates. Viral FeHV-1 DNA, but not infectious virus, was present in the optic nerve of one cat. However in this cat virus was isolated from the cornea making it difficult to conclude that the DNA present in the optic nerve was latent.

FeHV-1 was also detected within the ciliary, pterygopalatine, and cranial cervical autonomic ganglia examined in this study. Involvement of the autonomic ganglia has not previously been associated with FeHV-1 infection. In this study FeHV-1 established latent infections within the CCG of all cats. The consistent presence of latent FeHV-1 within the CCG is similar to the behavior of varicella zoster virus (VZV), also a member of the *Varicellovirus* genus*,* which is consistently found not only in cranial nerve ganglia [9,10], but also in autonomic systemic ganglia [12]. In contrast, HSV-1 latency in humans is restricted to cranial nerve ganglia [38,39]. Infection of the autonomic ganglia provides extensive neural connection from the nasal passages and oropharynx to the ocular surface and intra-ocular structures [40]. These neural pathways have been well described by Labetoulle [32]. Latency within the autonomic ganglia as well as the TG may results in herpetic ocular disease which could involve not only the cornea, but also the uveal tract and retina [41].

Histologically the CG examined all appeared normal. Therefore either inflammatory changes do not occur in the CG or they were very focal and missed on histological sectioning. In contrast, all of the TG collected at days 6 and 10 PI showed ganglionic cell death, depletion of ganglion cells, increased numbers of glial cells, and lymphoplasmacytic inflammation. In human patients with HSV-1 infection histologic examination of the ganglia has not been performed during active infection due to the obvious difficulty in obtaining tissue samples [42]. However in the murine model of HSV-1 infection, apoptosis of neurons and histiocytic and lymphocytic infiltrates have been noted [20-22]. In humans infected with VZV, neuritis and degeneration of sensory nerve roots have been noted [43,44]. In this study, only one section of TG from group 3 showed histologic changes: a loss of ganglion cells, but no concurrent inflammation. This was an unexpected finding as mice latently infected with HSV-1 typically demonstrate a mononuclear cellular infiltrate [23-27]. In human TG, latent HSV-1 infection has also been associated with chronic inflammation. A lymphocytic infiltrate has also been noted in bovine TG latently infected with bovine herpesvirus −1 (BHV-1) [45]. Interestingly in one study, lymphocytes consistently surrounded latently infected HSV-1 human TG neurons, but not the VZV-positive neurons [28]. Latent FeHV-1 infection may be more similar to latent VZV in this aspect.

In the current study, the FeHV-1 copy numbers in the TG were much higher than those previously reported [30]. This may be due to the fact that the time frame of this study was 30 days versus 56 days in the previous study. This may also be due to differences in the virulence of the viral strains used in each study, C-27 versus SGE [30]. The strain of HSV-1 has been shown to influence the number of latent virus particles within individual neurons in a murine model [46]. The amount of virus inoculated was higher in the previous study of FeHV-1 [30] than this study and therefore was unlikely to play a role in the increased copy numbers within the TG. Based on studies of HSV-1, latency is typically established in only a small percentage of sensory neurons in humans [47] and the copy number is usually only 2–50 copies/cell [47,48]. The copy numbers are reportedly similar for VZV [49,50]. Interestingly in our study wide variation was seen in the amount of latent FeHV-1 DNA detected despite all individuals receiving the same initial inoculum. In one study of a rabbit model of HSV-1, the number of viral genomes was directly proportional to the titer of infectious inoculum [51]. However in a different rabbit model and also murine model, this did not hold true [52,53]. In the study reported here the latent viral load was strongly associated with the severity of clinical signs as denoted by the total clinical score from day 0 to the day of euthanasia. For mice infected with HSV-1, a higher latency load is associated with an increased risk of recurrence [54]. In human patients with HSV-1 and HSV-2 infection, the severity of the first episode and the latency load are strongly linked to the recurrence rate [55]. This data would imply that animals with more severe clinical signs were at higher risk for recrudescence.

## Conclusions

The intra-ocular and neural involvement during FeHV-1 infection was more widespread than previously known. Autonomic ganglia as well as sensory ganglia harbored latent FeHV-1. The histologic response in neural tissue was similar to that seen during HSV-1 or VZV infection. Finally increased severity of clinical signs was associated with increased latent viral loads, likely increasing the risk of recrudescent infection.

## Methods

### Animals

All experiments were performed in full compliance with the ARVO Statement for the Use of Animals in Ophthalmic and Vision Research and with approval from the Michigan State University Animal Care and Use Committee. A total of fifteen 6-month-old female specific pathogen free (SPF) cats were obtained from a commercial vendor (Liberty Research, Inc, Waverly, NY). Cats were housed in individual cages with the infected and control groups in separate containment rooms (Biocontainment Level-2) with controlled temperature, humidity, and lighting. All personnel wore sterile disposable Tyvek coveralls, gloves, head covers, and shoes covers upon entering a containment room. Cats were fed a combination of dry and moist diets. All cats were acclimated for 7 days before virus exposure.

### Inoculation and clinical scores

Cats were anesthetized via chamber administration of halothane and oxygen. Twelve cats were infected by instilling 1.05 ml (1×10^5^ TCID_50_/ml) of the C-27 strain of FeHV-1 (ATCC, Manassas, VA) into the left and right conjunctival sacs (350 μl each), external nares (150 μl each), and oropharynx (50 μl). The other 3 cats served as controls. Animals were observed and clinical scores assigned daily to each cat by one of two trained observers after at least 15 minutes of observation using a scoring system modified from the USDA Supplemental Assay Method 311 (Table 3) [56].

**Table 3:** Clinical scoring system

**Clinical sign**	**Daily score if present**
**Fever**	
103.0 to 103.9°F	1
104.0 to 104.9°F	2
≥105°F	3
**Conjunctivitis/Ocular discharge**	1
**Rhinitis/Nasal discharge**	1
**Sneezing**	1
**Audible rales**	2
**Coughing**	2
**Open mouth breathing**	3
**Anorexia**	1
**Dehydration**	1
**Hypothermia < 99°F**	2
**Oral ulcers (lingual or oral mucosa)**	
1 ulcer <4 mm	1
Multiple ulcers <4 mm	3
Ulcer or ulcers >4 mm	5
**Salivating**	1
**External ulcers (lip or nares)**	
Non-bleeding ulcer	4
Bleeding ulcer	6

The following scoring system was modified from the published USDA Scoring Method for FeHV-1 [56] and was used to assign daily clinical scores for each cat. The highest possible score for a cat on any given day was 31.

### Serologic examination

All cats were determined to be serologically negative for FeHV-1 virus neutralizing antibodies prior to inoculation. Blood samples were collected for evaluation of FeHV-1 VN antibodies using a modified microtiter neutralization assay [57,58] on days 7, 14, and 21 post-inoculation from the cats euthanized on day 30 (group 3).

### Sample collection

Groups of 4 inoculated cats and 1 control cat were euthanized with an intravenous injection of pentobarbital sodium on day 6 PI when all FeHV-1 exposed cats were exhibiting ocular signs (group 1), on day 10 PI corresponding to peak ocular and respiratory signs (group 2), and on day 30 PI when the clinical signs had resolved and latency was established (group 3). Using sterile technique the entire right conjunctiva, cornea, uveal tract, retina, optic nerve, entire optic chiasm, CG, PTPG, CCG, TG, brainstem, visual cortex, cerebellum, and olfactory bulb were placed in individual Eppendorf tubes and frozen at –80°C. Gloves were changed and the instruments flamed with ethanol between each collection site. To each Eppendorf tube, 800 μl of Bovarnick’s solution and sterile homogenizing beads were added. The Eppendorf tube was then placed in the mixer mill (MM300, Qiagen, Valencia, CA) for tissue disruption and homogenization. The resulting homogenate was frozen at –80°C. Sections of the contralateral cornea and conjunctiva and the entire contralateral TG and CG were collected for histological evaluation.

### Virus isolation

A 200 μl aliquot of each tissue’s homogenate was reserved for DNA extraction. The remaining suspension was brought to 3 ml with Eagle’s Minimum Essential Medium (EMEM), supplemented with 10% fetal bovine serum. From the homogenate, 500 μl aliquots of filtered (0.45 μm) tissue extracts were inoculated onto monolayers of Crandell Reese feline kidney (CRFK) cells grown onto 24-well plates. The inoculated cells were examined daily for the appearance of characteristic cytopathic effects. Viral isolates were verified to be FeHV-1 by direct immunofluorescence (application of direct polyclonal FeHV-1 fluorescent antibody) (American BioResearch, Pullman, WA). Virus isolation attempts were performed in duplicate.

### SYBR green-based real-time quantitative PCR

DNA extraction was performed utilizing the Qiagen DNEasy Blood and Tissue kit (Qiagen, Valencia, CA). DNA quantitation was performed spectrophotometrically. Negative extraction controls consisted of 200 μl of PBS.

The real-time quantitative SYBR Green PCR assay targeted a 478 bp fragment containing the FeHV-1 gE gene. The sequence of the forward primer was 5′-GGT CAT GTG TAA TGT TGA CG-3′, and the sequence of the reverse primer was 5′- GTC TTT GGT TCT GAT GAG AG-3′ [56]. The sensitivity of this assay was 10 genomic copies of FeHV-1. Its dynamic range was confirmed by its ability to amplify the targeted DNA fragment of 48 known clinical isolates of FeHV-1.

The amplification kit used was the Quantitect SYBR Green PCR Kit (QIAGEN, Valencia, CA) with a final primer concentration of 0.5 μM. The 50 μl real time PCR reaction mixture consisted of 25 μl 2× SYBR Green mix (QIAGEN, Valencia, CA), 1 μl forward primer (25 pmol/μl), 1 μl reverse primer (25 pmol/μl), 100 ng of template DNA (20 ng of template DNA for CG and pterygopalatine ganglia) and ddH_2_O. Real-time PCR was performed on a iCycler™ iQ® System (Bio-Rad Laboratories, Hercules, CA) with the following cycling conditions: pre-denaturation at 95°C for 15 min, followed by 45 cycles of 94°C for 30 sec, 50°C for 30 sec and 72°C for 1 min. A post-amplification melt curve analysis was incorporated in the run to confirm the specificity of the amplicons generated. Post-PCR analysis was performed using the iCycler detection software version 1.1.

All reactions were conducted in duplicate. The DNA extraction controls and PCR reagent controls were included in each run. A standard curve of threshold cycle in relation to viral copy number was constructed. The standard curve for FeHV-1 was generated by a series of 10-fold dilutions (10^-1^ – 10^-6^ copies) of purified FeHV-1 stock DNA. Samples for the standard curve were assayed in triplicate for each run and also served as the positive control. As the number of FeHV-1 genomes/μg of ganglionic DNA was known, the FeHV-1 copy number per 100 cells was calculated based on 260,000 haploid genome equivalents/μg of feline cellular DNA [30].

### Histopathology

Following fixation in 10% buffered formalin, the conjunctiva, cornea, TG, and CG were paraffin-embedded, sectioned at a thickness of 5 μm, and stained with hematoxylin and eosin. Samples were examined by light microscopy by a single board-certified veterinary pathologist (MK).

### Statistical evaluation

Linear regression was performed to compare the total of the clinical scores from Day 0 to the day of euthanasia and the FeHV-1 copy number within the TG in groups 1, 2, and 3. Significance was set at p<0.05.

## Abbreviations

FeHV-1: Felid herpesvirus 1; BHV-1: Bovine herpesvirus 1; CCG: Cranial cervical ganglion; CG: Ciliary ganglion; HSV-1: Herpes simplex virus 1; PI: Post-inoculation; PTPG: Pterygopalatine ganglion; RT-PCR: Real time polymerase chain reaction assay; TG: Trigeminal ganglion; VI: Virus isolation; VN: Virus neutralizing; VZV: Varicella zoster virus.

## Competing interests

The authors declare that they have no competing interests.

## Authors’ contributions

WMT conceived of the study, participated in its design, coordinated the study, performed sample collection, performed the statistical evaluation, and drafted the manuscript. SJ assisted with study design, performed daily clinical scoring, performed sample collection, performed the RT-PCR, and helped to draft the manuscript. SHT assisted with study design, performed the virus isolation, and performed the RT-PCR. MK performed the histologic review of the specimens. AW assisted with study design, assisted with study coordination, assisted with virus isolation, and assisted with RT-PCR. RKM jointly conceived of the study, participated in its design, prepared the inoculum, oversaw the virus isolation, and oversaw the RT-PCR. All authors read and approved the final manuscript.

## Authors’ information

WMT and SJ are Diplomates of the American College of Veterinary Ophthalmologists. SHT completed his PhD in Comparative Medicine and Integrative Biology with a thesis entitled “*The complete and annotated genomic sequence of feline herpesvirus 1 (FHV-1) and an infectious BAC clone: a platform for studies of targeted mutants by recombineering.”* MK is a Diplomate of the American College of Veterinary Pathologists and serves as the head of the histopathology and immunohistochemistry laboratory at the Michigan State University Diagnostic Center for Population and Animal Health. AW is an academic specialist in virology. RKM is a professor in the department of microbiology and molecular genetics, serves as the section chief for virology, and has a specific research focus on FeHV-1.
